# Three-Dimensional Microscopy Characterization of Death Receptor 5 Expression by Over-Activated Human Primary CD4^+^ T Cells and Apoptosis

**DOI:** 10.1371/journal.pone.0032874

**Published:** 2012-03-06

**Authors:** Lucie Barblu, Jean-Philippe Herbeuval

**Affiliations:** CNRS UMR 8147, Université Paris Descartes, Paris, France; University of Nebraska Medical Center, United States of America

## Abstract

Activation-induced cell death is a natural process that prevents tissue damages from over-activated immune cells. TNF-Related apoptosis ligand (TRAIL), a TNF family member, induces apoptosis of infected and tumor cells by binding to one of its two death receptors, DR4 or DR5. TRAIL was reported to be secreted by phytohemagglutinin (PHA)-stimulated CD4^+^ T cells in microvesicles.

We investigate here TRAIL and DR5 regulation by activated primary CD4^+^ T cells and its consequence on cell death. We observed that PHA induced CD4^+^ T cell apoptosis in a dose-dependent manner. Thus, we investigated molecules involved in PHA-mediated cell death and demonstrated that TRAIL and DR5 were over-expressed on the plasma membrane of PHA-stimulated CD4^+^ T cells. Surprisingly, DR5 was constitutively expressed in naive CD4^+^ T cells at messenger RNA (mRNA) and protein levels. Thus, using 3 dimensional microscopy and intracellular staining assays, we show that DR5 is constitutively expressed in CD4^+^ T cells and is pre-stocked in the cytoplasm. When cells are stimulated by PHA, DR5 is relocalized from cytoplasm to plasma membrane. Small interference RNA (siRNA) and blocking antibody assays demonstrate that TRAIL/DR5 interaction is mainly responsible for PHA-mediated CD4^+^ T cell apoptosis. Thus, membrane DR5 expression leading to TRAIL-mediated apoptosis may represent one of the pathways responsible for eradication of over-activated CD4^+^ T cells during immune responses.

## Introduction

Elimination of over-activated immune cells is a natural process that prevents autoimmunity and damages of healthy organs. Activation–induced cell death (AICD) is one of the processes that contribute to eliminate over-activated T cells after immune response [1]. Apoptosis can be induced by interaction between death ligands and their death receptors [2], [3].

The Tumor Necrosis Factor (TNF) superfamily is composed by multiple apoptotic ligands, such as FasL [4], [5], tumor necrosis factor (TNF)-related apoptosis-inducing ligand (TRAIL) [6], [7], [8], and TNF-related weak inducer of apoptosis (TWEAK) [9] and their associated-receptors. TRAIL has been shown to induce apoptosis of the vast majority of tumor cell lines [10], [11] but does not kill normal cells [12]. This unique property is due to the fine regulation of TRAIL-mediated apoptosis by expression of two groups of receptors [13]. Three receptors do not induce apoptosis (Decoy Receptors, DcR) and two activate apoptosis of target cells (Death Receptor 4 and 5, DR4, DR5) [14], [15], [16]. The two biologically active forms of TRAIL, membrane-bound (mTRAIL) and soluble TRAIL (sTRAIL), are regulated by type I interferon (interferon-alpha and beta: IFN-α and IFN-β) [17], [18], [19]. DR4- and DR5-induced apoptosis activate the caspase pathway leading to apoptosis of target cells through the formation of a death inducing signaling complex (DISC) containing the death receptor and adaptor proteins such as Fas-associated death domain (FADD).

Previous reports showed that T cell blasts secreted bioactive forms of TRAIL and FasL in microvesicles shortly after Phytohemagglutinin (PHA) stimulation [20]. Following the release of these apoptotic ligands, T cells underwent apoptosis. The same group also demonstrated that CD8^+^ T cells were more susceptible to TRAIL regulation than CD4^+^ T cells. TRAIL regulation was defined by IL-2-dependent T cell growth in the absence of cell death induction, characterized by cell cycle arrest in G2/M [21]. Furthermore, the authors showed that PHA-induced T cell apoptosis was partially mediated by death receptors [22]. However, regulation of membrane expression of TRAIL death receptors following PHA-induced CD4^+^ T cell activation remains to be determined.

We provide here some new insight concerning death receptor 5 localization and regulation in primary CD4^+^ T cells. We show that DR5 is constitutively expressed in naive CD4^+^ T cells at messenger RNA (mRNA) and protein levels. Thus, using 3 dimensional (3D) microscopy assays, we demonstrate that DR5 is constitutively expressed in CD4^+^ T cells and is pre-stocked in the cell cytoplasm. Furthermore, under PHA stimulation, DR5 is relocalized to the plasma membrane. Small interference RNA (siRNA) and blocking antibody assays showed that TRAIL/DR5 interaction is largely responsible for PHA-mediated CD4^+^ T cells death. In contrast, T cell activation by anti-CD3/anti-CD28 antibodies induced plasma membrane expression of DR5 but not TRAIL. Consequently, this T cell activation does not lead to cell apoptosis due to the lack of TRAIL expression. Thus, membrane DR5 expression leading to TRAIL-mediated apoptosis may represent one of the pathways responsible for elimination of over-activated CD4^+^ T cells during immune response.

## Results

### PHA induced apoptosis of primary CD4^+^ T cells

**Figure 1:**
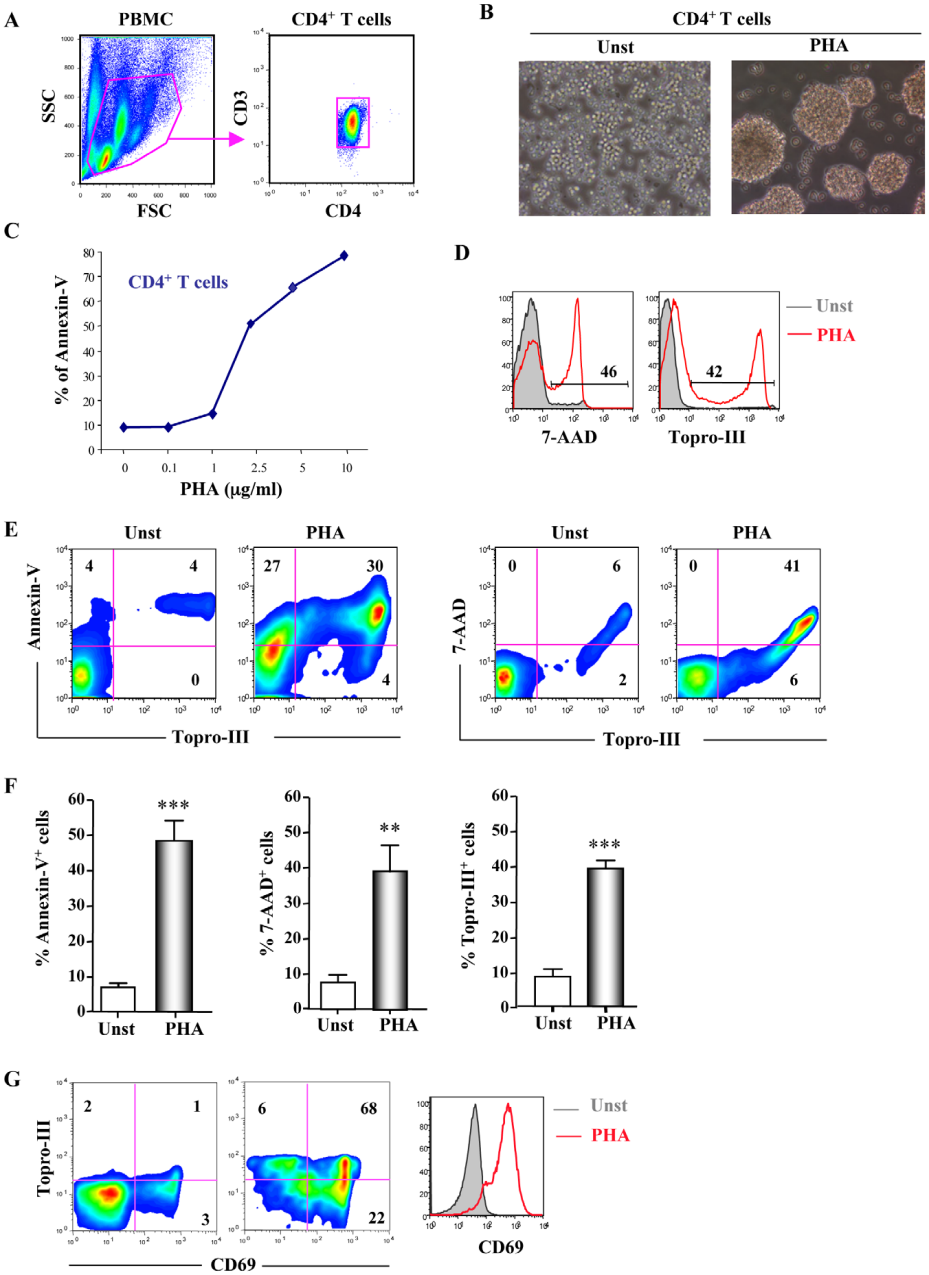
PHA induced Apoptosis of CD4^+^ T cells. **A.**CD4^+^ T cells were purified from PBMC from healthy donors. **B.** Cells were cultured 24 h in presence of 2.5 µg/ml of PHA and observed by microscopy (20×). PHA-treated cells are aggregated in multicellular blast. **C.** After 24 h, cells were analyzed by FACS. CD4^+^ T cells were cultured in presence or absence of increasing concentration of PHA and apoptosis was quantified by cytometry using Annexin-V staining. **D.** Apoptosis of CD4^+^ T cells cultured in presence or absence of 2.5 µg/ml was quantified using late (7-AAD, Topro-III) apoptotic markers by cytometry. **E.** Dot plots analysis of unstimulated or PHA-activated CD4^+^ T cells showing Annexin-V/Topro-III or 7AAD/Topro-III double staining. **F.** Percentage of Annexin-V, 7-AAD, Topro-III expressed by CD4^+^ T cells cultured in presence or absence of PHA for 24 h analyzed by cytometry. **G.** CD69 activation marker and Topro-III double staining of untreated or PHA-activated CD4^+^ T cells. P values (*p*) were determined using a two-tailed Student's *t* test comparing unstimulated versus PHA stimulation (p<0.05 one star, p<0.01 two stars, p<0.001 three stars). Data of panels **C**, **D**, **E** and **G** are representative of 4 independent experiments.

Primary CD4^+^ T cells were positively purified from fresh blood (Fig. 1A) and were cultured overnight in the presence or absence of PHA. We observed formation of pluricellular blastes in PHA-treated wells (Fig. 1B). We next studied apoptosis of PHA-activated CD4^+^ T cells by FACS using two different markers: Annexin-V, 7-AAD and Topro-III allowing us to discriminate between early (Annexin-V) and late apoptosis (7-AAD, Topro-III). We first tested several concentrations of PHA to induce T cell death overnight (Fig. 1C). 1 µg/ml of PHA was sufficient to induce low levels of apoptosis (15%), and 2.5 µg/ml induced more than 50% of Annexin-V positive CD4^+^ T cells. Between 2.5 µg/ml and 10 µg/ml of PHA we observed a dose dependent induction of Annexin-V by CD4^+^ T cells. Then, we performed late marker of apoptosis stainings (Topro-III and 7-AAD) on activated CD4^+^ T cells cultured overnight in presence of PHA at 2.5 µg/ml. PHA induced dramatic apoptosis of CD4^+^ T cells after 18 h of culture (Fig. 1D). We observed that apoptotic cells expressing Topro-III were also 7-AAD positive, confirming that cells were in the late stage of apoptosis (Fig. 1E and F). In contrast, we observed that the vast majority but not all CD4^+^ T cells expressing Annexin-V were Topro-III positive (Fig. 1E and F). Because Annexin-V is an early marker of apoptosis, cells expressed Annexin-V before Topro-III, which is a late marker of apoptosis. Thus, we tested whether apoptotic cells were activated, using the CD69 T cell activation marker. The vast majority of CD69 positive cells were also expressing Topro-III, confirming that over-activated CD4^+^ T cells underwent apoptosis (Fig. 1G).

### TNF family member expression by PHA-activated CD4^+^ T cells

**Figure 2:**
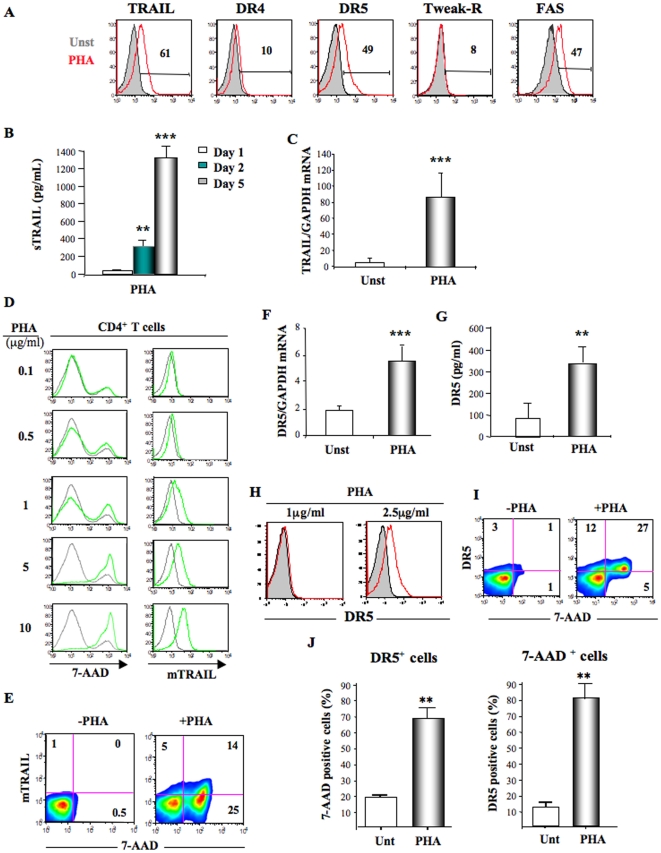
TNF family member expression by PHA-activated CD4^+^ T cells. **A.** CD4^+^ T cells from healthy donors were cultured 24 h in presence (red histograms) or absence (solid grey histograms) of PHA (2.5 µg/ml) and were phenotyped for TRAIL, DR4, DR5, Tweak-R and Fas expressions by FACS. **B.** Quantification of soluble TRAIL production by ELISA of PHA-activated CD4^+^ T cells after 1, 2 and 5 days of culture. **C.** TRAIL mRNA quantification of unstimulated or PHA-activated CD4^+^ T cells. mRNA levels were analyzed by quantitative RT-PCR. Results are expressed as the ratio of TRAIL to GAPDH gene reporter. **D.** CD4^+^ T cells were cultured 24 h in presence of increasing concentration of PHA. Cells were stained with 7-AAD and membrane TRAIL (mTRAIL) antibody and were analyzed by flow cytometry. DR5 mRNA quantification of unstimulated or PHA-activated CD4^+^ T cells. **E.** Dot-Plot analysis of 7-AAD and mTRAIL stainings. Cells were cultured in absence or presence of PHA (2.5 µg/ml) and stained with 7-AAD and membrane TRAIL antibody. **F.** mRNA levels were quantified by quantitative RT-PCR. Results are expressed as the ratio of DR5 to GAPDH gene reporter. **G.** Quantification of DR5 content in PHA activated CD4^+^ T cells after 24 h of culture. Cells were lysed and DR5 levels in lysates were quantified by ELISA. **H.** FACS analysis of DR5 membrane expression by primary CD4^+^ T cells stimulated with 1 µg/ml and 2.5 µg/ml of PHA (red histograms) compared to untreated CD4^+^ T cells (grey). **I.** Dot plot cytometry analysis of resting or PHA-activated CD4^+^ T cells showing 7-AAD and DR5 expression. **J.** CD4^+^ T cells were cultured in absence or presence of PHA for 24 h. Graphics show the percentage of 7-AAD positive cells among DR5 expressing cells under, and proportion of DR5+ among 7-AAD expressing cells. Data of panels **A**, **D**, **E**, **H** and **I** are representative of 4 independent experiments. P values (*p*) were determined using a two-tailed Student's *t* test (p<0.05 one star, p<0.01 two stars, p<0.001 three stars).

We next investigated the pathway involved in PHA-mediated apoptosis. CD4^+^ T cells were tested for TRAIL, DR4, DR5, Tweak-R and Fas expression. Overnight stimulation with PHA induced strong cell surface expression of TRAIL, DR5 and Fas, while DR4 and Tweak-R were not upregulated by CD4^+^ T cells (Fig. 2A).

Thus, we studied TRAIL and DR5 regulation by primary CD4^+^ T cells. PHA activation had been demonstrated to induce soluble TRAIL secretion, which participated to T cell activation [20]. CD4^+^ T cells were cultured 1-to-5 days in the presence or absence of PHA. We observed significant soluble TRAIL secretion after 2 and 5 days (Fig. 2B), while 18 h were sufficient to induce membrane TRAIL expression (Fig. 2A). We then studied TRAIL mRNA expression. Cells were cultured 18 h in the presence or absence of PHA. After extraction, mRNA was quantified by quantitative RT-PCR and expressed in ratio TRAIL/GAPDH. PHA induced very high levels of TRAIL mRNA expression compared to untreated CD4^+^ T cells (Fig. 2C). Furthermore, we observed a positive parallel between 7-AAD and TRAIL expression, both increasing simultaneously with PHA concentration (Fig. 2D). It should be noted that the majority of TRAIL expressing cells (75%) are also 7AAD positive. However, the majority of 7-AAD positive cells are TRAIL negative (Fig. 2E).

TRAIL induces apoptosis by binding to one of its two death receptors DR4 or DR5 [15]. DR4 was lightly upregulated by PHA, while DR5 expression was strongly increased on CD4^+^ T cell membrane (Fig. 2A). For this reason, we focused our research on DR5 study. mRNA study by quantitative RT-PCR revealed that DR5 mRNA levels in CD4^+^ T cells were highly increased by PHA. Surprisingly, DR5 mRNA was also expressed in non-activated CD4^+^ T cells, suggesting a constitutive expression of DR5 (Fig. 2F). To verify whether previous mRNA levels were traduced into protein, we quantified by Elisa DR5 protein expression after cells lysis. DR5 protein was increased by PHA, and significant constitutive levels of DR5 protein was observed in non-activated CD4^+^ T cells (Fig. 2G). FACS analysis of extracellular staining revealed that DR5 was not expressed at the plasma membrane when low dose of PHA was used (1 µg/ml) to stimulate CD4^+^ T cells (Fig. 2H). We showed above that this concentration was too low to induce apoptosis (Fig. 1C). In contrast, higher dose of PHA (2.5 µg/ml) was inducing membrane DR5 expression (Fig. 2H) and apoptosis of CD4^+^ T cells (Fig. 1C). These latest results strongly suggested that presence of DR5 was responsible for apoptosis induction.

Finally, to link DR5 expression and CD4^+^ T cell apoptosis, we performed flow cytometry analysis. CD4^+^ T cells were cultured overnight in absence or presence of PHA (2.5 µg/ml) and cells were stained with 7-AAD, Annexin-V and DR5 antibodies. Dot-plots analysis revealed that the majority but not all DR5 positive cells also expressed 7-AAD (Fig. 2I). The vast majority of DR5 expressing CD4^+^ T cells also expressed 7AAD (69%±6) and the vast majority of 7-AAD expressing cells were also DR5 positive (81%±9) (Fig. 2J). This demonstrates that membrane DR5 expression is directly linked to CD4^+^ T cell apoptosis.

### Microscopic study of DR5 expression in CD4^+^ T cells

**Figure 3:**
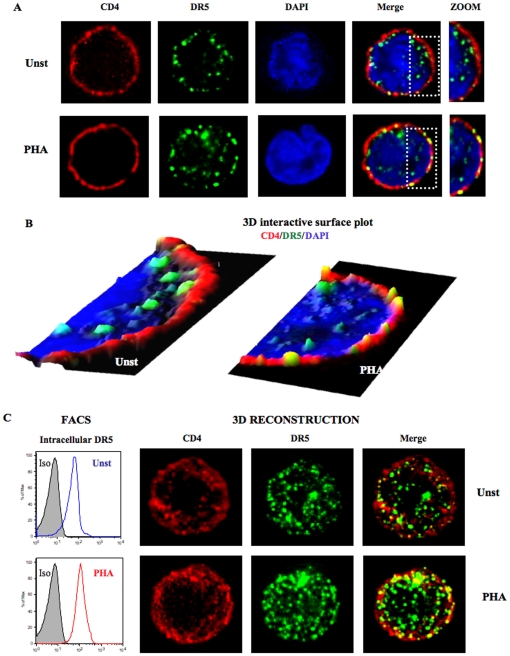
3D microscopy study of DR5 expression by CD4^+^ T cells. **A.** CD4^+^ T cells were cultured overnight in presence or absence of PHA (2.5 µg/ml) and stained for DR5 (green), nuclear staining DAPI (blue) and CD4 (red) to study DR5 protein expression and localization. Unstimulated CD4^+^ T cells exhibited DR5 protein expression in the cytoplasm at close membrane proximity, defined by CD4 staining (upper panels). In contrast, PHA-activated CD4^+^ T cells expressed intracellular and membrane DR5. DR5/CD4 colocalization appears in yellow. To better visualize DR5 localization, a zoom of the dotted line defined zone from CD4/DR5/DAPI overlay picture was added (extreme right panels). **B.** DR5 analysis using 3D interactive surface plot. Previous 3D microscopy pictures were analyzed using 3D interactive surface plot. Representation allowed a clear visualization of intracellular DR5 (green) and membrane CD4 (red). Unstimulated CD4^+^ T cells harbored only intracellular DR5 inside the CD4 (membrane) delimitation. PHA stimulation of CD4^+^ T cells induced the export of DR5 protein at the surface delineated by CD4 staining (red) but some DR5 also remained intracellular. Yellow spots represent DR5/CD4 colocalization. **C.** FACS analysis of intracellular staining of DR5 and compilation of all plans from previous microscopic images were used to perform 3D reconstruction. This representation illustrates the difference of DR5 quantity between unstimulated and activated CD4^+^ T cells. PHA-stimulated cells expressed more DR5 (green) and show more colocalization (yellow) with CD4 than unstimulated cells. Each picture is representative of the vast majority of the observed cells on the slides.

For a complete study of DR5 expression and localization we developed 3D microscopic experiments. Purified CD4^+^ T cells were cultured overnight in the presence or absence of PHA (2.5 µg/ml). To visualize the nucleus, cells were stained with DAPI and anti-CD4 antibody (red) to localize the plasma membrane. DR5 protein (green) was detected using DR5 monoclonal antibody. As illustrated in Figure 3A, image plane analysis showed that CD4 (red) was homogeneously expressed and precisely delineated CD4^+^ T cell plasma membrane. Microscopic analysis revealed that CD4^+^ T cells from healthy donor expressed intracellular but no or very low extracellular DR5 (green). DR5 was essentially localized in the cytoplasm, delineated by the membrane (CD4, red) and the nucleus (DAPI, blue). DR5 was located in close proximity to the membrane but remained in the cytoplasmic compartment (Fig. 3A). This result confirmed that DR5 is constitutively expressed in resting CD4^+^ T cells as mRNA experiments suggested (Fig. 2E). Microscopy images clearly show that PHA-activated CD4^+^ T cells expressed high levels of DR5 protein at their CD4-delineated membrane (Fig. 3A), consistent with FACS analysis (Fig. 2A and F).

To better visualize DR5 localization in CD4^+^ T cells, 3D surface plot and 3D reconstruction analyses were performed (Fig. 3B–C). 3D interactive surface plot using ImageJ software allowed us to distinguish with precision the internal from external localization of DR5 (Fig. 3B). We clearly observed that DR5 remained essentially in cytoplasm in non-activated cells, and relocalized at the plasma membrane when stimulated by PHA. 3D reconstruction was used to illustrate the difference of DR5 quantity between unstimulated and PHA-activated CD4^+^ T cells (Fig. 3C). 3D reconstruction represents a compilation of all images (stacks) analyzed for one cell. This representation shows a global view of the total quantity of each protein tested. 3D reconstruction images revealed that PHA-stimulated cells expressed more DR5 and exhibit more colocalization with CD4 (yellow) than untreated cells (Fig. 3C). These results were confirmed by intracellular staining that showed higher total DR5 expression in PHA-stimulated than in unstimulated CD4^+^ T cells (Fig. 3C). 3D surface plot and reconstruction combined analysis clearly demonstrated that PHA-stimulated CD4^+^ T cells harbored plasma membrane DR5 expression in contrast to the restrictive intracellular DR5 expression of unstimulated CD4^+^ T cells.

**Figure 4:**
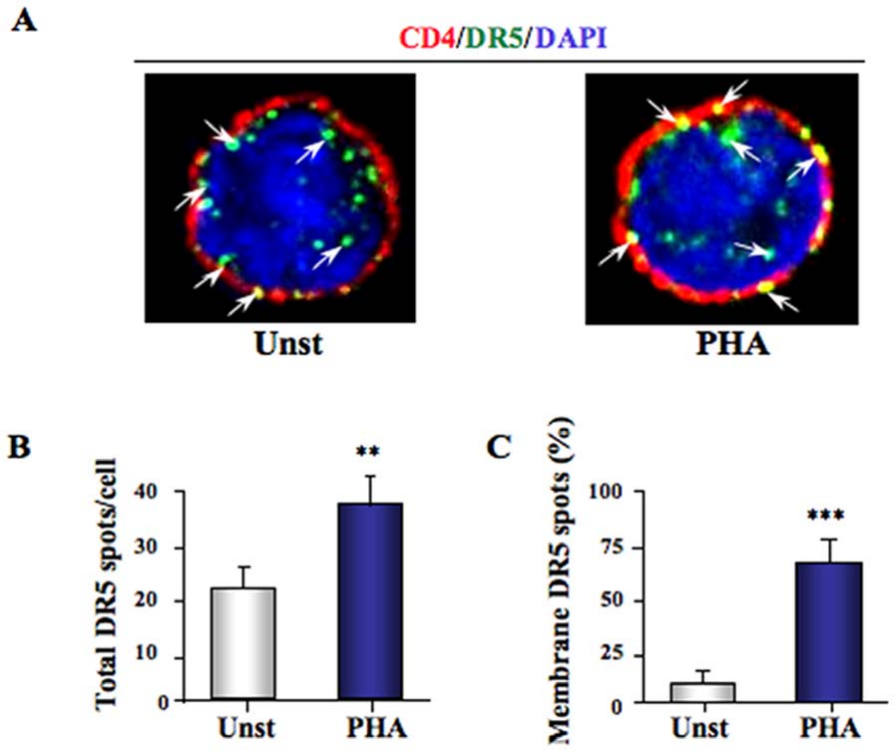
3D microscopy quantification of DR5 expression by CD4^+^ T cells. **A.** CD4^+^ T cells were cultured overnight in presence or absence of PHA (2.5 µg/ml) and stained for DR5 (green), nuclear staining DAPI (blue) and CD4 (red) to study DR5 protein expression and localization. 10 cells per experiment were quantified for their DR5 spot number in 3 independent experiments for a total of 30 cells per condition (untreated or PHA-stimulated). White arrows show some examples of the DR5 spots that were counted **B.** Total number of DR5 spots in 30 untreated and 30 PHA-stimulated cells. Results show the number of DR5 spots per cell **C.** Percentage of membrane DR5 expression in 30 untreated and 30 PHA-stimulated cells. P values (*p*) were determined using a two-tailed Student's *t* test (p<0.05 one star, p<0.01 two stars, p<0.001 three stars).

Finally, we quantified DR5 expression in untreated and PHA-activated CD4^+^ T cells by 3D microscopy (Fig. 4). DR5 expression was quantified by counting the green and yellow spots (DR5 that colocalized with CD4) in 30 untreated and 30 PHA-stimulated cells (3 independent experiments) (Fig. 4A). We found that the total number of DR5 spots was increased by PHA compared to untreated cells (p = 0.002) (Fig. 4B). This result is in accordance with the increased DR5 mRNA and DR5 protein that we previously quantified by quantitative RT-PCR and Elisa, respectively (Fig. 2F and 2G). Furthermore, the number of DR5 spots on the membrane was also increased by PHA stimulation. Untreated CD4^+^ T cells expressed very low membrane DR5 (less than 5% of the DR5 spots are on the membrane) contrasting with PHA-stimulated CD4^+^ T cells which expressed more than 60% of DR5 protein on their membrane (p = 0.001) (Fig. 4C).

### Inhibition of PHA-mediated CD4^+^ T cell apoptosis

**Figure 5:**
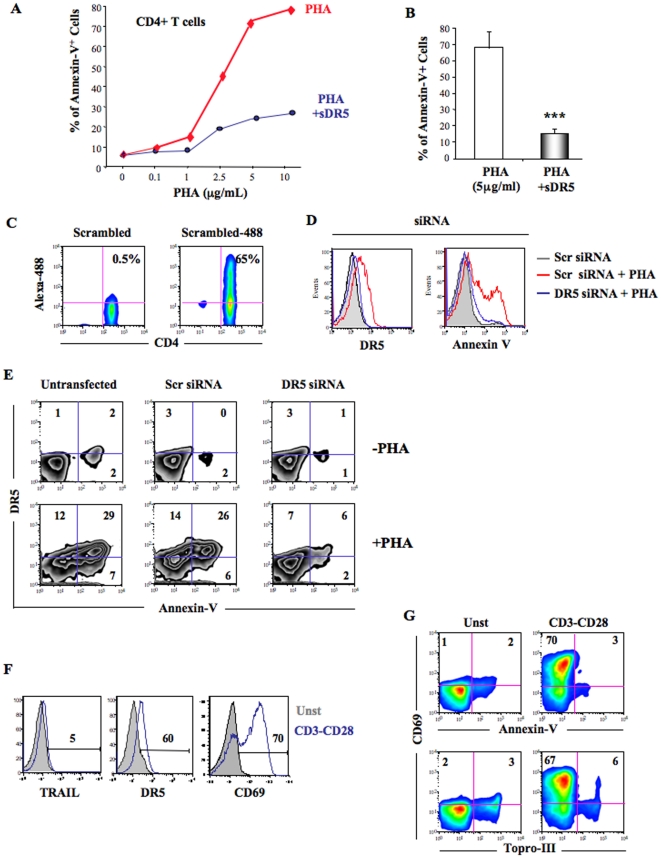
Apoptosis inhibition of CD4^+^ T cells by soluble DR5 and DR5 siRNA. **A.** CD4^+^ T cells were cultured 24 h in presence of increasing concentration of PHA and with or without 5 µg/ml of DR5. Apoptosis was quantified using Annexin-V staining by flow cytometry. **B.** CD4^+^ T cells were cultured 24 h in presence of 5 µg/ml of PHA and with or without soluble DR5 (5 µg/ml). Apoptosis was quantified using Annexin-V staining by flow cytometry. **C.** Efficiency of siRNA transfection in CD4^+^ T cells. Cells were transfected with unlabelled or Alexa-488 scrambled siRNA overnight. Dot plots show transfection efficiency of CD4^+^ T cells by Scr-Alexa-488 siRNA (right panel). **D.** Transfection of CD4^+^ T cells with DR5 siRNA significantly inhibited DR5 expression by PHA-stimulated CD4^+^ T cells in contrast to scrambled (Scr) siRNA (left panel). Inhibition of DR5 expression at the surface of PHA-stimulated CD4^+^ T cells using DR5 siRNA also dramatically inhibited Annexin-V positive cells induced by PHA compared to Scr siRNA (right panel). **E.** Dot plot analysis of untransfected and transfected CD4^+^ T cells with scrambled siRNA (Scr siRNA), DR5 siRNA (DR5 siRNA). After overnight transfection, CD4^+^ T cells were cultured in absence (upper panels) or presence (lower panels) of PHA. Images show dot plots of DR5/Annexin-V staining. **F.** TRAIL, DR5 and CD69 FACS analysis by CD4^+^ T cells stimulated overnight in presence of anti-CD3 and anti CD28 antibodies. **G.** FACS dot plot analysis of CD69-Annexin-V and CD69-Topro-III staining of CD4^+^ T cells cultured in presence or absence of anti-CD3/CD28 activation overnight. Data of panels C, D, E, F and G are representative of 4 independent experiments. P values (*p*) were determined using a two-tailed Student's *t* test (p<0.05 one star, p<0.01 two stars, p<0.001 three stars).

Finally to definitively link TRAIL/DR5 expression with CD4^+^ T cell apoptosis, we inhibited the TRAIL pathway using either the TRAIL blocker soluble DR5 (sDR5) or silencing RNA (siRNA) techniques. Recombinant soluble DR5 is a natural blocker of TRAIL-mediated apoptosis that binds to soluble and/or membrane TRAIL. CD4^+^ T cells were cultured with several concentrations of PHA and in presence or absence of sDR5 (5 µg/ml). As shown in Fig. 5A, sDR5 dramatically reduced CD4^+^ T cell apoptosis even when high doses of PHA (5 and 10 µg/ml) were used in culture. Figure 1B clearly showed that sDR5 at 5 µg/ml significantly inhibited induction of Annexin-V expression by PHA-activated CD4^+^ T cells (Fig. 5B).

To better demonstrate the role of DR5 in PHA-mediated cell death, we performed DR5 silencing mRNA (siRNA) experiments. Freshly isolated CD4^+^ T cells were transfected with scrambled or DR5 siRNA overnight. The percentage of live transfected cells was quantified using Alexa-488 labeled siRNA. More than 65% of CD4^+^ T cells were efficiently transfected (Fig. 5C) by scrambled siRNA. We show that CD4^+^ T cells transfected with DR5 siRNA harbored massive downregulation of DR5 expression compared to scrambled transfected cells (Fig. 5D). Furthermore, DR5 siRNA transfected CD4^+^ T cells become resistant to PHA-induced apoptosis (Fig. 5E). We demonstrate here that the inhibition of DR5 expression by siRNA dramatically reduced PHA-mediated apoptosis confirming a central role of TRAIL/DR5 in cell death of activated CD4^+^ T cells.

Finally, we tested whether another kind of T cell activation would induce TRAIL pathway and apoptosis. CD4^+^ T cells were stimulated using anti-CD3/CD28 antibodies. FACS analysis revealed that anti-CD3/CD28 activation induced DR5 but not TRAIL expression on plasma membrane of primary CD4^+^ T cells. Anti-CD3/CD28 antibodies activated the vast majority of CD4^+^ T cells (70%) (Fig. 5F). However, we did not observe apoptosis even of activated cells. T cell activation by those antibodies induced expression of DR5 and CD69 on plasma membrane but not TRAIL, which likely accounts for the lack of T cell apoptosis. (Fig. 5G).

## Discussion

TRAIL is a very promising candidate for immune-based therapy due to its sophisticated way of inducing apoptosis. TRAIL does not exhibit cytotoxic effects on normal cells and tissues [12] and is potentially efficient to eradicate a large panel of cancer cells [11], [23]. Several clinical trials are currently evaluating TRAIL anti-tumor effect, alone or in combination with other chemotherapeutic drugs [24], [25], [26], [27], [28], [29] [30], [31]. TRAIL is also involved in several pathologies including HIV-1 [32], [33]. Indeed, plasma TRAIL has been reported to be an early pathogenic marker in acute HIV-1 infection and is correlated to viral load in chronic disease [34], [35]. HIV-1 upregulates DR5 expression on the membrane of CD4^+^ T cells *in vitro* [32], [33] making them prone to TRAIL-mediated apoptosis [36]. Thus, understanding regulation of TRAIL and its receptors by human immune cells may have important physiologic relevance.

Soluble TRAIL and soluble FasL produced in supernatants of PHA-activated PBMC have been shown to induce apoptosis of Jurkat T cell line [37]. Furthermore, TRAIL and FasL were reported to be sometimes localized into the same vesicular compartment, but they are sometimes secreted separately. TRAIL secretion by T cells was detected very soon after high dose of PHA (50 µg/ml) exposure [37]. Although TRAIL secretion had been well documented, DR5 localization and expression remained to be clarified.

Our results show that massive cell death occurs when cells were cultured for 24 h in presence of PHA, similar to an earlier study [37]. We demonstrate using early and late apoptotic markers that PHA-induced apoptosis of CD4^+^ T cells was proportional to PHA concentration. Cells that underwent apoptosis were mostly activated cells (CD69^+^). Thus, we clearly show a parallel between activation and apoptosis.

We further investigated the pathway by which PHA-induced apoptosis occurs. Our analysis of TNF family members revealed that TRAIL and DR5 were highly upregulated on CD4^+^ T cell membranes compared to those of unstimulated cells. As previously reported, CD4^+^ T cells could also produce [20] soluble TRAIL after several days in culture with PHA. However, overnight stimulation was not sufficient to activate soluble TRAIL secretion in contrast to membrane TRAIL, which was rapidly expressed after stimulation. These soluble and membrane forms of TRAIL might be differently regulated. We also tested whether the lack of soluble TRAIL production after overnight culture could be due to a rapid mobilization by membrane DR5 highly expressed by activated cells. We cultured CD4^+^ T cells with PHA and anti-DR5 antibodies, and measured sTRAIL production (data not shown). The addition of DR5 blocking antibodies did not increase sTRAIL production. Thus, the apoptosis observed after overnight PHA activation could not be due to soluble TRAIL or soluble FasL. We also observed that the majority but not all CD4^+^ T cells expressing DR5 were apoptotic. This could be explained by the fact that DR5 has to be expressed on plasma cell membrane and then followed by TRAIL binding leading to apoptosis. Thus, it is not surprising that we detected DR5-expressing cells that did not yet interact with TRAIL-expressing cells and consequently were not into apoptotic stage.

DR5 protein distribution in human primary cells has not been well characterized. Therefore, our most surprising results concerned DR5 expression in primary CD4^+^ T cells. We show here that DR5 mRNA and protein are constitutively expressed in freshly purified CD4^+^ T cells. Flow cytometry analysis of intracellular DR5 staining uniquely revealed a constitutive expression of DR5 in primary CD4^+^ T cells confirming Elisa results. This constitutive DR5 expression by human immune T cells has not been previously described. Therefore, we developed a 3D microscopy model to better characterize DR5 protein localization in primary CD4^+^ T cells. Microcopy study revealed that resting CD4^+^ T cells expressed intracellular stock of DR5. These results were in accordance with mRNA and Elisa data. Death receptors are constitutively expressed and stocked in cytoplasm of CD4^+^ T cells. Thus, after cell activation DR5 could be rapidly mobilized at the cell surface. These unexpected results suggest that CD4^+^ T cells are “programmed” to be eliminated by apoptosis shortly after strong activation. In fact, we report that DR5 is relocalized from cytoplasm to membrane under PHA stimulation. However, it should be noticed that increased DR5 expression by PHA-stimulated CD4^+^ T cells could also be due to increased expression of total DR5 protein. Thus, PHA induces membrane DR5 relocalization and increased of DR5 level in T cells. Images from microscopy show membrane expression of DR5 and colocalization with plasma membrane CD4 after PHA exposure.

Finally, we clearly demonstrated that TRAIL/DR5 pathway was the major inducer of PHA-activated CD4^+^ T cell apoptosis after a short period of culture (less than 24 h). We first observed a correlation between plasma membrane DR5 expression and levels of apoptosis. Soluble DR5 significantly reduced CD4^+^ T cells apoptosis. To definitively link TRAIL-DR5 interaction and apoptosis, we performed siRNA experiments. Silencing DR5 dramatically reduced PHA-mediated CD4^+^ T cell apoptosis demonstrating that TRAIL-DR5 pathway is largely involved in PHA-induced cell death. However, it should be noted that inhibition of the TRAIL pathway did not totally reduce cell death, suggesting that other pathways are involved, such as Fas/FasL [38]. Furthermore, Fas/FasL may also contributes to T cell apoptosis after longer period of activation (48 h).

Silencing mRNA experiments also demonstrated that inhibition of DR5 mRNA largely reduced DR5 membrane expression after PHA stimulation. This indicates that DR5 mRNA over production is necessary for high levels of membrane DR5 expression.

Interestingly, anti-CD3/CD28 T cell activation, which is a more physiological stimulation than PHA, induced DR5 expression but not TRAIL on plasma membrane of CD4^+^ T cells. Consequently, we did not observe apoptosis of CD3-CD28 activated T cells. In this setting, the limiting factor is TRAIL and not DR5. Thus, we could imagine that DR5 represents a functional T cell activation marker, and that activated DR5^+^CD4^+^ T cells undergo apoptosis depending on physiological conditions. Membrane TRAIL expression may be regulated by strong non-physiological PHA stimulation or by type I interferon (IFN), which are secreted by plasmacytoid dendritic cells (pDC) after viral infection, such as HIV-1 or influenza A infection [39], [40]. Thus, apoptosis of CD4^+^ T cell in viral context could be dependent on the ability of virus to induce DR5 expression in CD4^+^ T cells. HIV-1 specifically activates CD4^+^ T cells and induces DR5 membrane expression after contact of cellular CD4 and viral gp120 [33]. In parallel, TRAIL expression is upregulated by type I IFN produced by HIV-activated pDC, leading to TRAIL-DR5-mediated CD4^+^ T cell apoptosis [40]. The AIDS syndrome is characterized by massive CD4^+^ T cell depletion, mainly due to the TRAIL pathway [34], [41]. In contrast, influenza virus also activates IFN-α production by pDC that induces TRAIL expression on CD4^+^ T cells [39], [42]. However, influenza does not activate DR5 expression on CD4^+^ T cells, and TRAIL-DR5-mediated apoptosis of CD4^+^ T cells is not observed. Thus, membrane DR5 expression appears to be a key factor regulating the balance between primary CD4^+^ T cell apoptosis and survival.

In conclusion, we demonstrate that strong T cell activation leads to rapid TRAIL-DR5-mediated apoptosis of human primary CD4^+^ T cells. This fast cell death could be explained by the rapid mobilization of DR5 protein from the cytoplasm stocks, observed in non-activated CD4^+^ T cells, to the plasma cell membrane when cells are strongly stimulated. This mechanism of activation-induced cell death (AICD) of human primary T lymphocytes is rapid, and thus may have physiological relevance.

## Materials and Methods

### Patients blood samples

Blood from 30 healthy HIV-1-seronegative blood bank donors was obtained from “Etablissement Français du Sang” (convention # 07/CABANEL/106), Paris, France.

### Ethics Statement

Experimental procedures with human blood have been approved by Necker Hospital Ethical Committees for human research and were done according to the European Union guidelines and the Declaration of Helsinki.

#### Isolation and culture of blood leukocytes

*In vitro* experiments were performed using peripheral blood mononuclear cells (PBMC) isolated by density centrifugation from peripheral blood leukocyte separation medium (Cambrex, Gaithersburg, MD). CD4^+^ T cells were purified using the CD4 purification kit (Miltenyi Biotech, Bergisch Gladbach, Germany). Cells were cultured in RPMI 1640 (Invitrogen, Gaithersburg, MD) containing 10% fetal bovine serum (Hyclone, Logan, UT) and 1% Pen-Strep-Glut (Invitrogen).

#### PHA stimulation

CD4^+^ T cells from healthy donors were seeded at 50,000 cells per 100 µl and cultured overnight with several PHA concentrations (ranging form 0.1 to 10 µg/ml). T cell activator anti-CD3 and anti-CD28 (BD Biosciences, San Jose, CA) antibodies were coated 1 h at 37°C in PBS 1× on plastic wells were used to activate CD4^+^ T cells. Supernatants were collected for cytokine detection and cells were used for flow cytometry (FACS) experiments.

#### Apoptosis assay

After 24 h of culture with PHA, purified CD4^+^ T cells were washed with Annexin buffer (BD Bioscience) and incubated 15 minutes with Annexin-V (BD Bioscience), Topro-III (Invitrogen) and 7-ADD (BD Bioscience) at 4°C. Soluble recombinant DR5 (Myltenyi) was used at several concentration (0.1–10 µg/ml) to block PHA-induced apoptosis. Apoptosis was quantified and analyzed by multicolor flow cytometry.

#### Flow cytometry

CD4^+^ T cells were incubated for 20 min at 4°C with Vioblue-conjugated anti-CD4 (Miltenyi), FITC-DR5 (eBioscience, San Diego, CA), PE-TRAIL (eBioscience), Tweak-R-PE (R&D system), Fas-APC (BD Bisoscience), CD69-FITC (BD Bioscience) or with appropriate isotype-matched control antibodies (at 5 µg/mL each) in PBS containing 2% mouse serum (Sigma, Saint Louis, MO). Cells were washed twice in ice-cold PBS-FBS 2% and FACS analysis was performed on a FACSCanto 7 colors flow cytometer using FACSDiva software (BD Biosciences). FlowJo software (Treestar, Ashland, OR) was used to analyze flow cytometry data.

#### Cytokine detection

Purified CD4^+^ T cells were cultured in presence or absence of PHA from 1 to 5 days. Supernatants were collected and tested for their soluble TRAIL content using TRAIL ELISA kit (Diaclone). For DR5 protein quantification, stimulated or non stimulated CD4^+^ T cells were cultured overnight and total cells were lysed. Lysate DR5 content were quantified using DR5 Elisa Kit (Invitrogen).

#### siRNA T cell transfection

CD4^+^ T cells were seeded at 10^5^ cell/100 µl in 96 well plates and incubated at 37°C. 3 µl of Hiperfect transfection reagent (Qiagen, Valencia, CA) were added to appropriate siRNA concentration and adjusted at 100 µl with serum-free medium. Then, the solution was gently mixed and incubated at room temperature during 30 minutes. After incubation, the mix was added to cells in culture. Finally, cells were incubated at 37°C overnight with or without PHA (2.5 µg/ml). Control was performed using scrambled siRNA and transfection efficiency was tested by FACS using Alexa-488 tagged scrambled siRNA (all from Qiagen). Apoptosis was determined using Annexin-V staining and analyzed by flow cytometry.

#### RNA extraction

500 µL of Trizol were added to the pellet of CD4^+^ T cells the cells were then mixed during 1 minute and incubated at room temperature during 5 min. 100 µL of chloroform were added and the mix were centrifuged at 13 000 rpm during 15 min. The upper phase were recovered and 500 µL of isopropanol were added and mixed during 1 hour at 4°C. The mix were then centrifuged during 10 min at 13 000 rpm. 500 µL of 75°C ethanol were added to the pellets after isopropanol were removed (13 000 rpm during 15 min). Finally, the pellets were recovered in H_2_0-DEPC. The RNA were stored at −80°C.

#### Reverse transcription

The quantity of RNA was dosed and 1 µg of RNA per sample was used to perform the reverse transcription. We prepared mix of 12 µL including the mRNA, hexa-primers and H_2_0-DEPC. The samples were incubated at 65°C during 5 mins. A mix containing 5× Buffer, dNTP Mix, RT enhancer and Verso Enzyme was prepared (Verso cDNA Kit, Thermo Fisher Scientific, abgene, UK). The samples were incubated 5 min at room temperature, 1 hour at 42°C and then 2 min at 95°C.

#### PCR

Expression of DR5 gene by CD4^+^ T cells was developed. Primers were synthesized by Invitrogen (primer DR5 sens 5′ AGGTGAAGTGGAGCTAAGTC 3′, DR5 antisens: 5′ TCACTCCAGGGTGTACAATC 3′; primer β2-microglobuline sens: 5′ CCAGCAGAGAATGGAAAGTC, β2-microglobuline antisens GATGCTGCTTACATGTCTCG). Amplification reaction were realized with a Mastercycler Eppendorf machine according to the following program (Tm 55°C): Lid 110°C, 5 min at 94°C, 1 min at 55°C and 1 min 30 at 72°C, 1 min at 94°C (35 cycles) and finally 10 min at 72°C. Samples were run on an eletrophoresis gel agarose 1% during 2 h at 80 V. The threshold level was determined by the software according to the optimization of the standard curve. Arbitrary quantity values were assigned to the resulting standard and 4-fold serial dilutions were made to obtain 8-point standard curve. Results are presented as ratios between the target gene and the GAPDH mRNA.

#### Three-dimensional microscopy

Purified CD4^+^ T cells were cultured overnight in presence of PHA. CD4^+^ T cells were plated on poly-L-lysine (Sigma)-coated slides and then fixed in 4% paraformaldehyde, quenched with 0.1 M glycine. Cells were incubated in permeabilizing buffer containing 1% saponin with mAb DR5 (ebioscience) and with Alexa547 labeled anti-CD4 (BD bioscience). DR5 staining was revealed by a goat anti-mouse IgG-Alexa488 (Jackson ImmunoResearch, West Grove, PA). Nucleus was stained using DAPI (Molecular Probes, Paisley, UK). Mounted slides were scanned with a Nikon Eclipse 90i Upright microscope (Nikon Instruments Europe, Badhoevedorp, The Netherlands) using a 100× Plan Apo VC piezo objective (NA 1.4) and Chroma bloc filters (ET-DAPI, ET-GFP) and were subsequently deconvoluted with a Meinel algorithm and 8 iterations and analyzed using Metamorph® (MDS Analytical Technologies, Winnersh, UK). DR5/CD4/DAPI/Overlay/Confocal plane: Representative 2D focal plan. XZ/YZ view of Confocal plane: XZ/YZ projection of the XY focal plan along the red cross axis. Overlay with bright: DIC. Reconvolution overlays: 2D projections of the maximum intensity pixels along the Z axis. 3D: 3D reconstruction analysis of the total cell using the ImageJ64 software (NIH, Bethesda, MD, USA).

#### Statistical analysis

Experiments were repeated at least four times. P values (*p*) were determined using a two-tailed Student's *t* test. *p*<0.05 was considered statistically significant. Unvaried distributions of flow cytometric data were performed by probability binning, in 300 bins using FlowJo software [43].
